# Supplementary material for: Monitoring the Early Strength Development of Cement Mortar with Piezoelectric Transducers Based on Eigenfrequency Analysis Method
Source: Sensors (Basel). 2022 Jun 2;22(11):4248. doi: 10.3390/s22114248 (PMC9185401; doi:10.3390/s22114248)
Supplement: Supplementary file 1 [file sensors-22-04248-s001.zip › sensors-1727757-supplementary.pdf]

sensors

Supplementary Materials

Monitoring the Early Strength Development of Cement Mortar with Piezoelectric Transducers Based on Eigenfrequency Analysis Method

Guocheng Wang [1], Wenying Qiu [1], Dongkai Wang [1], Huimin Chen [2], Xiaohao Wang [1,2] and Min Zhang [2,*]

[1] Tsinghua-Berkeley Shenzhen Institute, Tsinghua University, Shenzhen 518055, China; wgc19@mails.tsinghua.edu.cn (G.W.); qwy17@mails.tsinghua.edu.cn (W.Q.); wdk18@mails.tsinghua.edu.cn (D.W.); wang.xiaohao@sz.tsinghua.edu.cn (X.W.)
[2] Shenzhen International Graduate School, Tsinghua University, Shenzhen 518055, China; chenhm19@mails.tsinghua.edu.cn
* Correspondence: zhang.min@sz.tsinghua.edu.cn

Supplementary Materials for this manuscript include the following:

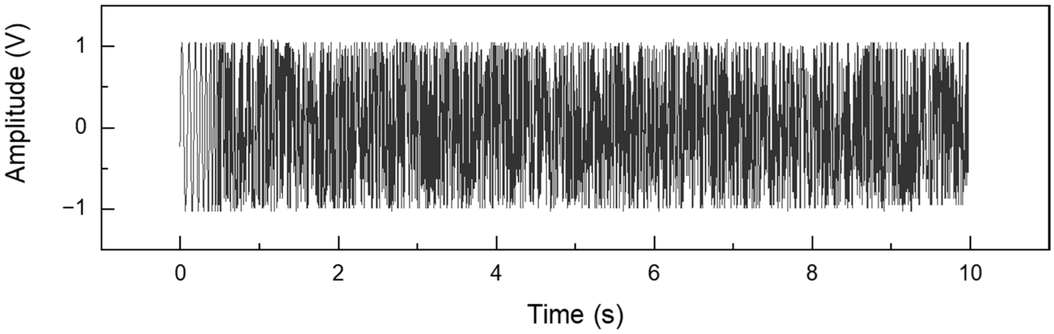

Figure S1. The excitation sweep signal from 10 Hz to 200 kHz generated by the signal generator.

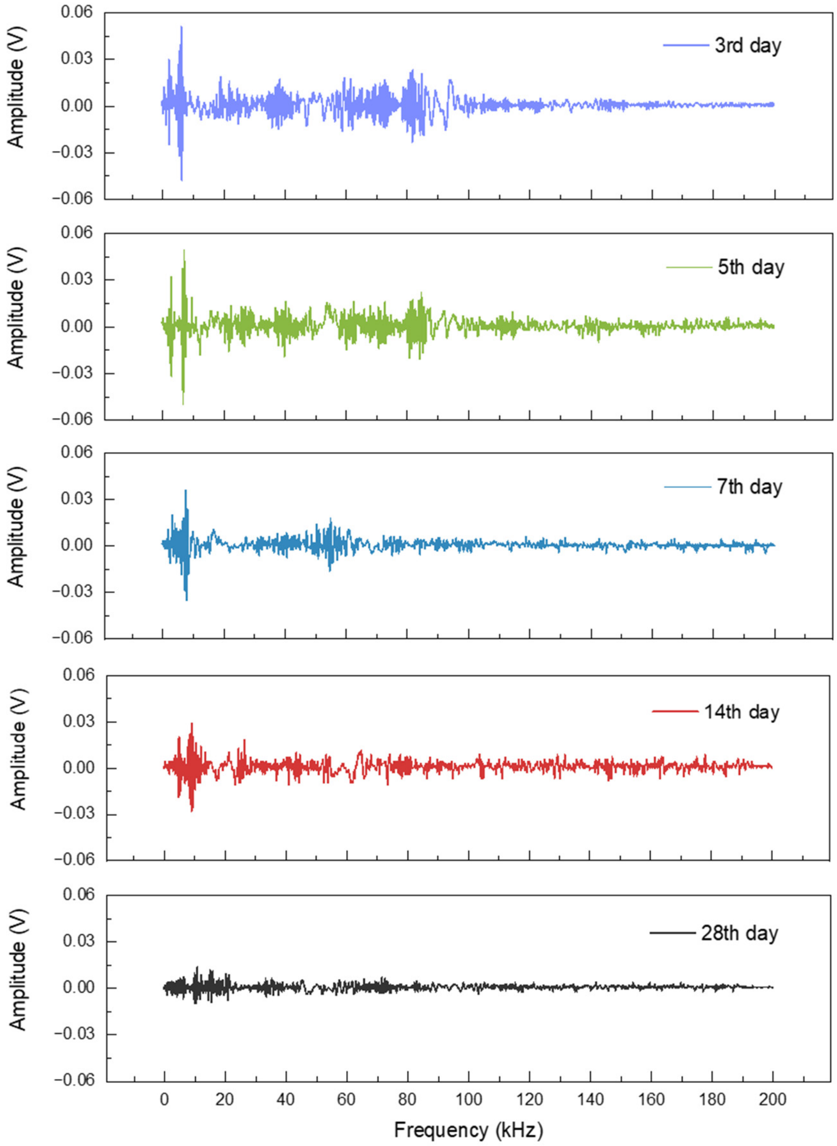

Figure S2. The received signal of P.S 32.5 specimens with swept sine excitation at 3rd day, 5th day, 7th day, 14th day, and 28th day.

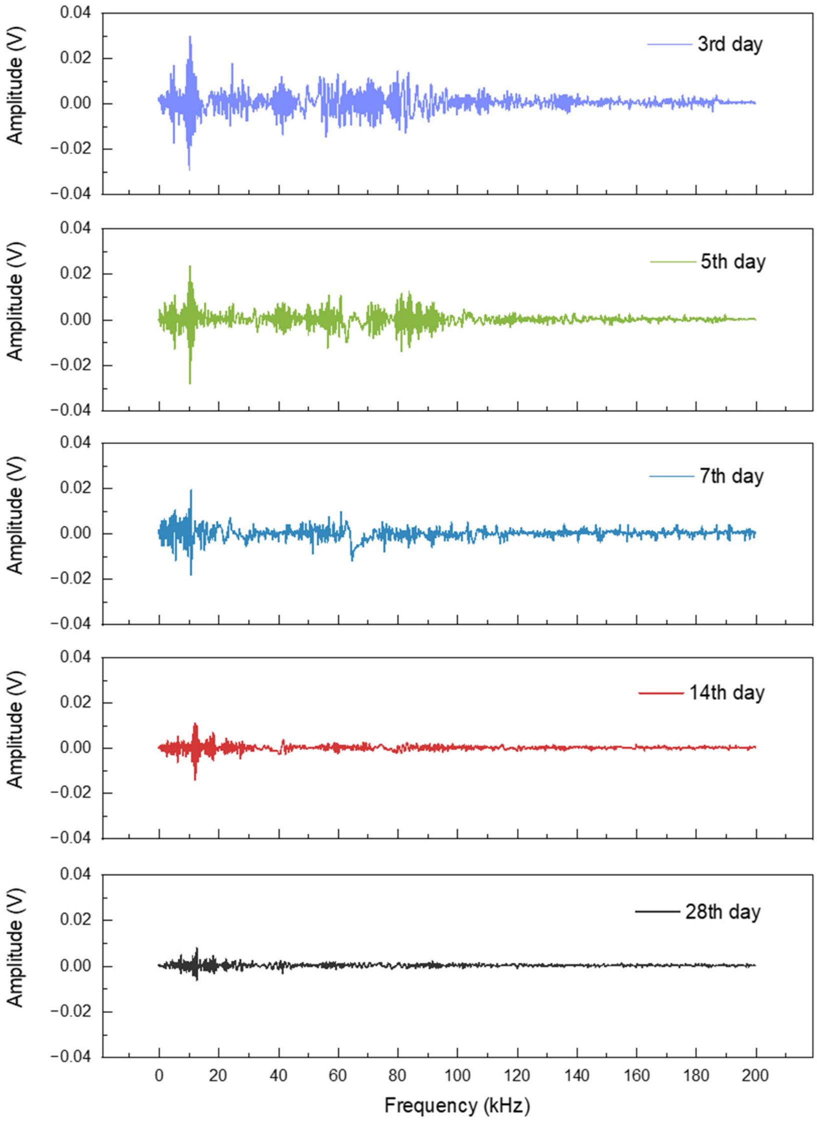

Figure S3. The received signal of P.O 52.5 specimens with swept sine excitation at 3rd day, 5th day, 7th day, 14th day, and 28th day.

Table S1. Setting parameters of materials in simulation.

Materials	Density (10^3 kg/m^3)	Young's Modulus (GPa)	Poisson Ratio
PZT-5H	7.45	46	0.33
Cement mortar P.S 32.5	2.63	30	0.30
Cement mortar P.O 42.5	2.63	32.5	0.30
Cement mortar P.O 52.5	2.63	34.5	0.30
Epoxy resin	1.18	24	0.38
